# Advances and controversies in acute decompensated heart failure treatment: beta-blocker roles, emerging devices, and future directions

**DOI:** 10.1097/MS9.0000000000003592

**Published:** 2025-07-16

**Authors:** Fathi Milhem, Omar Almur, Orabi Hajjeh, Mohammad Bdair, Abdelfattah M. Dahmas, Karol B. Haddad, Abdalhakim Shubietah, Osama S. Al-Said, Rama Al-Braik, Maram M. Abukhalil, Qutayba Z. Ayaseh, Hammam Jallad, Lina Karaki, Husam Hamshari, Mohammed AbuBaha

**Affiliations:** a Faculty of Medicine and Health Sciences, An-Najah National University, Nablus, Palestine; b Faculty of Medicine, Arab American University, Jenin, Palestine; c Internal Medicine Department, Advocate Illinois Masonic Medical Center, Chicago, Illinois; d Faculty of Medicine, Jordan University of Science and Technology, Irbid, Jordan; e Faculty of Medicine, Al-Quds University, Jerusalem- Abu Dies, Palestine

**Keywords:** acute decompensated heart failure (ADHF), biomarker-guided treatment, emerging therapies, management strategies, pathophysiology

## Abstract

Acutely decompensated heart failure (AHF) is a severe, multifactorial syndrome with acute symptom worsening, which poses a great challenge for healthcare professionals worldwide. AHF admissions are responsible for a high percentage of morbidity, mortality, and healthcare utilization, particularly in elderly comorbid patients. The pathophysiology, clinical presentation, and treatment of AHF are presented in this review, emphasizing neurohormonal activation, hemodynamic derangements, and comorbidities such as chronic kidney disease, chronic obstructive pulmonary disease, and atrial fibrillation. Diagnostic and therapeutic approaches, including the use of beta-blockers, inotropes, and developing device-based therapies, are addressed. Controversies central to the discussion are the balancing of relief of symptoms with the possibility of adverse effects of high-dose inotropes and diuretics, the contentious continuation of beta-blockade in acute decompensation, and the emerging fluid management techniques, including ultrafiltration and the use of SGLT2 inhibitors. Also featured are promising advances in the areas of biomarker-directed therapies, regenerative medicine, and RNA-based therapies. New devices and telemedicine platforms are some of the emerging technologies underlining the shift toward precision medicine and multidisciplinary care. Despite progress being made, AHF is a heterogeneous and developing field, and further research and innovation are necessary. The intersection of pharmacological innovation, personalized medicine, and digital health offers new possibilities for the enhancement of outcomes, demanding collaboration among clinicians, researchers, and policymakers in addressing the ongoing challenges of this high-burden state.

## Graphical abstract

**Figure:**
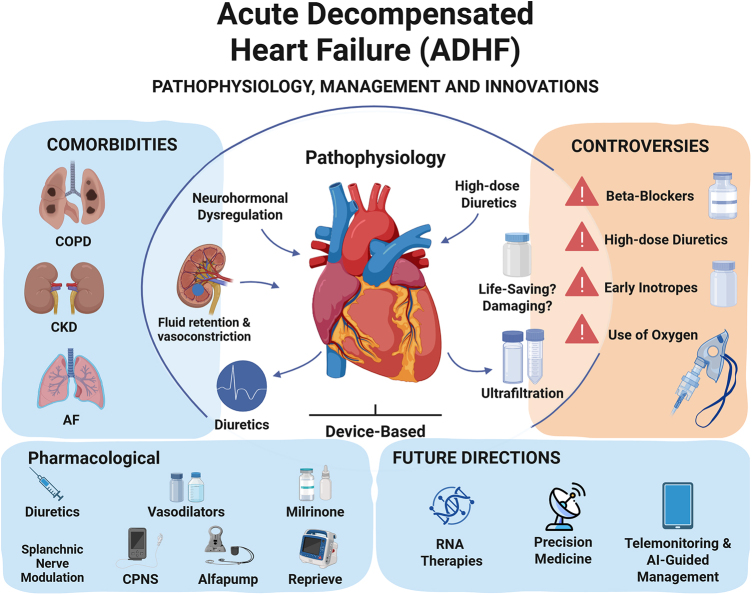

## Introduction

Heart failure (HF) is a major health problem worldwide, affecting several million individuals and contributing to enormous healthcare costs. Acute exacerbations, also known as acute decompensated heart failure (ADHF), are critical events clinically manifested by an acute worsening of symptoms that often necessitate immediate medical intervention^[1,2]^. These exacerbations significantly contribute to morbidity and mortality among patients with HF; thus, an update on comprehensive knowledge and strategies of management is warranted^[3,4]^. Acute HF exacerbation is notably disabling, considering the frequency at which it occurs and the number of poor outcomes associated with it^[5]^. Statistics highlight these events’ profound impact on patient outcomes and healthcare resources. HF remains the number one admitting diagnosis in adults over the age of 65 years and is responsible for over one million hospital admissions per year in the United States alone^[6,7]^. Rates of readmission within 30 days of discharge remain painfully high, with nearly 25% of HF patients needing prehospitalization^[8]^. These repeated hospitalizations add to the ever-increasing cost of healthcare and bring to the fore the pressing need for better interventions, which will reduce their frequency and severity^[9]^.

The consequences of acute HF exacerbations extend beyond the limits of direct medical care and touch on quality of life, functional capacity, and even long-term survival^[10]^. These events constitute a critical turn in the course of the disease, further modification of therapeutic strategies being needed, with intensified vigilance in clinical monitoring at every turn. With a greater percentage of the population aging and the management of chronic diseases, the prevalence of HF continues to rise^[11]^. Not surprisingly, societal and economic burdens from acute exacerbations have similarly increased with growing prevalence. Adding further complicating factors, the typical emotional and psychological consequences on the patient and caregivers continue to underline the multi-dimensional nature of the challenges these acute events pose. Current research and forming statistical trends show the necessity to meet this growing healthcare challenge with innovative solutions^[12,13]^.

For example, acute exacerbations of HF are not only common but also a major determinant of healthcare resource utilization: patients with acute HF often need treatments in the intensive care unit (ICU) and prolonged days in the hospital^[14]^. Based on these hospitalizations, combined with the burden of viable management of comorbid conditions, it is clear that multidisciplinary approaches to care and the need for therapeutic advances truly are paramount^[15]^. Finally and most importantly, inequalities in access to care and disparities in treatment approaches provide a further impetus for the need for uniform and equitable management approaches across different healthcare settings^[16]^. One of the more controversial aspects of acute HF management relates to the role of beta-blockers during exacerbations. Whereas beta-blockers remain one of the cornerstones of chronic HF treatment, their use during periods of acute decompensation remains controversial. Indeed, various indications support the continuation of beta-blockers in certain scenarios, whereas potential hemodynamic instability and contraindications raise several concerns in others^[17,18]^. In essence, understanding the organization of such controversies and implications for clinical practice is a core determinant in making clinical decisions on both inpatient and outpatient management strategies^[19]^. This review aims to give a detailed discussion of the exacerbations of acute HF, including types, underlying mechanisms, and management guidelines as they currently stand.

Concomitantly, this review will discuss the available pharmacologic and non-pharmacologic therapies for these critical events and address the current controversies in and emerging therapeutic strategies for the care of patients with HF, including the debated role of beta-blockers. In trying to aid decision-making, where consensus exists and debate is ongoing for clinicians, researchers, and healthcare professionals in an attempt to improve patient outcomes in HF, this synthesis of current evidence/discussion will be presented. It will ultimately lead to better understanding and inform clinical decision-making; thus, it will act as a catalyst for novel strategies in the management of acute exacerbations in HF patients. This review further provides original novelty by comprehensively integrating conventional therapies with evolving innovations such as investigational device-based strategies, precision medicine, and RNA-based therapeutics – areas that are underrepresented in existing literature. It also uniquely synthesizes the clinical implications of AI-driven care and telemedicine in the ADHF management pathway, making it a forward-looking and practically relevant reference for the modern clinician. Established practices and areas of ongoing investigation will both be explored in this review, with the hope of aiding a broader effort toward the advancement of care for one of the most challenging and impactful conditions in modern medicine.

## ADHF: pathophysiology, clinical profiles, and management

### Types of acute HF exacerbations

HF is subdivided into two main types: *de novo* acute HF and acute decompensated HF ADHF. *De novo* AHF occurs when there is a sudden increase in intracardiac filling pressures, often triggered by events like ischemia, leading to reduced peripheral perfusion and pulmonary edema. On the other hand, ADHF typically affects patients with preexisting cardiomyopathy or chronic HF^[20]^. ADHF has recently been redefined as a condition distinct from AHF, though it was previously considered the same. As defined, it is the rapid exacerbation of previous congestive HF, caused by a decline in cardiac function that leads to new signs and symptoms of congestion and poor organ perfusion as a complication of overwhelmed compensatory mechanisms of chronic heart failure (CHF), which requires urgent hospitalization^[21,22]^. The CHF compensatory mechanism maintains adequate tissue perfusion and is regulated by neurohormonal response. Neurohormonal activation in ADHF, involving the renin-angiotensin-aldosterone system (RAAS) and the sympathetic nervous system (SNS), initially compensates for reduced cardiac output but later becomes maladaptive, these neurohormonal activations create a feedback loop that drives vasoconstriction and fluid retention, further straining the heart and worsening ADHF symptoms^[23]^, leading to fluid accumulation overload, and organ congestion. Diuretics and vasodilators used to mitigate volume overload further activate these mechanisms, contributing to continued decompensation. In addition, sympathetic stimulation due to dysregulated neurohormonal responses to tissue hypoxia can cause transient vasoconstriction in the splanchnic and peripheral venous circulation, redistributing fluid into the pulmonary circulation, which worsens acute decompensation episodes^[24]^. Patients with ADHF can be categorized into four hemodynamic profiles based on the presence of congestion (elevated left-sided filling pressures or pulmonary capillary wedge pressure [PCWP]) and perfusion status (cardiac output or cardiac index [CI]). In the warm and dry profile (CI >2.2, PCWP <18 mmHg), patients exhibit stable hemodynamics without signs of congestion or hypoperfusion, requiring no immediate intervention. The warm and wet profile (CI >2.2, PCWP >18 mmHg) is marked by congestion, such as dyspnea, edema, or jugular venous distention, and is managed with decongestive therapies like diuretics. The cold and dry profile (CI <2.2, PCWP <18 mmHg) reflects hypoperfusion without congestion, presenting symptoms like fatigue, oliguria, or cold extremities, and often necessitates careful fluid resuscitation or inotropic support. The most severe profile, cold and wet (CI <2.2, PCWP >18 mmHg), involves both hypoperfusion and congestion, requiring a balanced approach to relieve congestion and improve perfusion with diuretics, vasodilators, or inotropes. Proper identification of these profiles through clinical assessment guides targeted treatment strategies to optimize outcomes^[23]^.

**HIGHLIGHTS**

- Acute decompensated heart failure (ADHF) is a significant health care problem with multifactorial pathophysiology that plagues humans worldwide.
- The review defines newer diagnostic methods and classification of ADHF, such as hemodynamic profiles and comorbidity interactions.
- Use of beta-blockers in acute decompensation is rigorously reviewed with focus on up-to-date evidence and areas of clinical debate.
- Emerging device-based therapies and non-pharmacologic innovations are explored, including ultrafiltration and neuromodulation systems.
- Future directions in managing ADHF focus on integrating biomarker-guided therapy, precision medicine, and digital health resources.

### Comorbidities and risk factors

**Figure 1.**
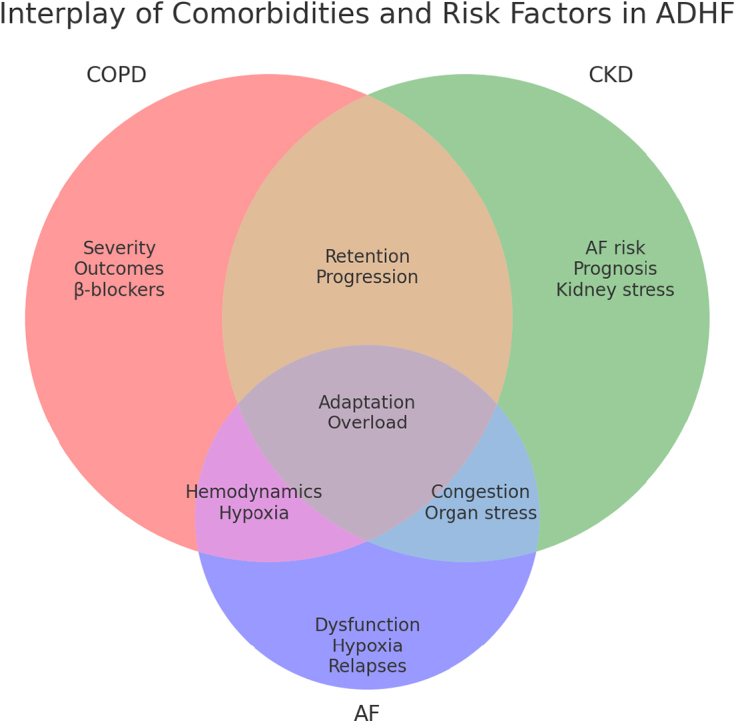
Venn diagram of comorbidities and risk factors in ADHF. This Venn diagram illustrates the interplay between key comorbidities – chronic obstructive pulmonary disease (COPD), chronic kidney disease (CKD), and AF – and their contributions to the progression of ADHF. Overlapping areas highlight shared effects, such as fluid retention, hypoxia, and organ congestion, while individual sections emphasize unique impacts of each condition. The diagram underscores the complexity of managing ADHF in patients with multiple comorbidities.

The clinical manifestation of ADHF is significantly influenced by comorbidities and risk factors. According to recent research, patients with ADHF frequently present with coexisting disorders, and chronic obstructive pulmonary disease (COPD) is particularly prevalent among patients with ADHF and relates to more severe disease progression and poorer outcomes. This overlap is significant because COPD affects nearly one-third of HF patients; the concurrent presence of COPD complicates HF management, especially with therapies such as β-blockers. COPD patients with ADHF may have limited tolerance to β-blockers due to their potential to exacerbate airway obstruction (Fig. 1)^[23]^. Alongside COPD, there is another prevalent condition in ADHF patients, the development of new-onset acute kidney injury (AKI) or the progression of previous AKI to acute kidney disease AKD. ADHF can lead to AKI, especially as a result of poor cardiac output and reduced renal perfusion^[25,26]^. This specific interaction between the heart and kidneys dysfunction is termed type 1 cardio renal syndrome CRS, where impairment in one organ exacerbates the other. Persistent AKI is linked to worse outcomes^[27]^. AKD, a condition representing a progression or incomplete recovery from an AKI episode, patients both with and without a prior AKI could be diagnosed with AKD, indicating its potential to develop independently in the context of ADHF. In patients hospitalized with ADHF, studies have shown that AKD develops in approximately 39.4% of those with an initial AKI episode and in about 19.4% without an initial AKI diagnosis, indicating that ADHF itself predisposes patients to AKD (Fig. 1)^[24,28]^. In addition, cirrhosis represents a significant comorbidity in patients with ADHF, Cirrhosis presents a challenge in the management of ADHF due to its complex effects on patient disease progression^[29]^. The presence of cirrhosis in ADHF patients is associated with increased mortality, prolonged hospital stays, key complications of cirrhosis, hepatorenal syndrome, ascites, and hepatic encephalopathy, which contribute to worsened outcomes, further complicating the clinical picture^[30,31]^. Moreover, atrial fibrillation (AF) is strongly associated with ADHF due to several interrelated mechanisms. In ADHF, AF often develops because of left ventricular dysfunction, excessive catecholamine release, acute hypoxia, and other factors like hypokalemia and sympathomimetic agents (Fig. 1)^[32]^. While most cases of in-hospital AF resolve spontaneously as HF improves, AF occurring after discharge is associated with poorer outcomes, including an increased risk of rehospitalization, cardiac death, and overall mortality^[1]^. Post-discharge AF recurrence often involves asymptomatic episodes and prolonged durations before resolution, which contribute to electrical and structural remodeling of the atria, potentially leading to persistent or chronic AF^[33]^. Furthermore, chronic kidney disease (CKD) is an independent risk factor for AF recurrence, adding complexity to the clinical prognosis^[34]^. Acute infections, particularly respiratory infections, are a common cause of decompensation, accounting for approximately 28%–29% of cases internationally^[35,36]^. Infections increase metabolic demand and physiologic stress, which can exceed the respiratory capacity in patients with significant left ventricular dysfunction, worsening ADHF symptoms^[37]^. Patients with severe symptoms, such as respiratory distress, hypoxemia, marked hemodynamic instability, or toxic appearance, require hospital admission due to high short-term mortality^[38]^. COVID-19 infection has emerged as an additional risk factor, with mortality rates reaching up to 25% in HF patients requiring admission. This highlights the need for emergency clinicians to consider these factors and recognize when admission is necessary for managing high-risk patients with acute decompensated HF^[39]^. In addition to these comorbidities, multiple risk factors independently contribute to ADHF progression and severity (Fig. 1). These factors, including hypertension, diabetes, ischemic heart disease, and lifestyle-related behaviors such as smoking, noncompliance with medication, and high salt intake, significantly increase the risk of hospitalization and poor prognosis. Understanding the interplay between comorbidities and these precipitating factors is essential for effective patient management, emphasizing the need to consider both aspects during clinical assessment and treatment planning^[40]^.

### Pathophysiology of ADHF

**Figure 2.**
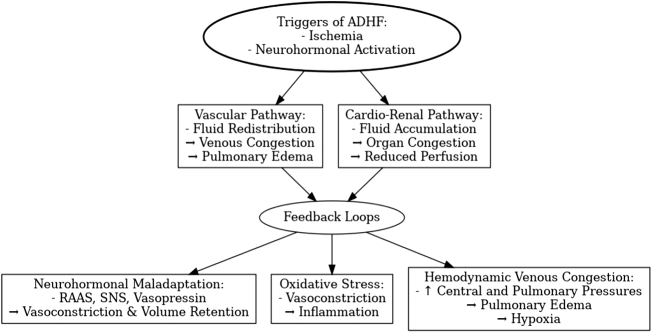
Detailed pathophysiology of ADHF: triggers, pathways, and feedback loops. This figure illustrates the detailed pathophysiology of ADHF. The process begins with triggers such as ischemia and neurohormonal activation, leading to two primary pathways: the vascular pathway, involving fluid redistribution and pulmonary edema, and the cardio-renal pathway, resulting in fluid accumulation, organ congestion, and reduced perfusion. Feedback loops, including neurohormonal maladaptation, oxidative stress, and hemodynamic venous congestion, exacerbate the condition through mechanisms like vasoconstriction, volume retention, inflammation, and hypoxia.

The pathophysiology of ADHF involves a series of hemodynamic instability, includes two primary pathways: a vascular (fluid redistribution) pathway and a cardio renal (fluid accumulation) pathway, as recognized by the European Society of Cardiology, and neurohormonal changes^[1,41]^, both compromise the heart’s ability to pump enough blood for whole body, which causes a cascade of cardiac, renal, and vascular dysfunctions, leading to fluid retention and tissue congestion^[42]^. Conventional views hold that congestion results from worsening heart function. Studies suggest that venous congestion and fluid buildup are not only symptoms but also active drivers of ADHF; conventional wisdom maintains that congestion is the outcome of deteriorating heart function^[43]^. There is evidence linking these pathways, with venous congestion leading to tissue edema and endothelial strain, which in turn triggers oxidative stress and the production of vasoconstrictors^[44]^. Furthermore, venous distension triggers sympathetic activation and inflammation. Small shifts in venous volume, which houses over 70% of blood, can significantly impact cardiac filling pressures, creating a cycle where fluid accumulation and vasoconstriction intensify each other (Fig. 2)^[45]^.

#### Hemodynamic venous congestion

Acute decompensated HF is a complex disorder characterized by diffuse hemodynamic disturbances. The core pathophysiological event involves severe venous congestion. Venous congestion triggers neurohormonal excitations, endothelial dysfunction, and renal dysfunction. This sets a vicious circle of fluid overload, myocardial stress, and progressive cardiac dysfunction in motion^[22]^. Fluid redistribution from peripheral or splanchnic venous systems to the central circulation commonly occurs, with increased central venous and pulmonary pressures. This heightens ventricular wall stress, compromises myocardial oxygenation, and worsens diastolic dysfunction, especially in a patient with a preexisting history of HF, preserved or reduced ejection fraction^[46]^. The rise in atrial and venous pressures greatly increases the pulmonary capillary hydrostatic pressure, forcing fluid leakage into the lungs through a familiar mechanism, giving way to pulmonary edema. Hypoxia further deteriorates this condition by depressing cardiac contractility and forming a self-sustaining vicious cycle of decompensation^[47]^. Compensatory mechanisms, such as fluid retention mediated by the RAAS activation and neurohormonal vasoconstriction, attempt to restore cardiac output but not infrequently further increase venous congestion and raise afterload^[48]^. It is also due to the persistence of fluid retention and high filling pressure that contribute to mitral regurgitation, thereby further worsening pulmonary and systemic congestion, further deteriorating renal function and hemodynamic stability (Fig. 2)^[49]^.

#### Neurohormonal pathway

In ADHF, neurohormonal activation plays a critical role in worsening symptoms through mechanisms of fluid retention and vasoconstriction. Key systems, including the RAAS, the SNS, and vasopressin, drive volume expansion and arterial and venous vasoconstriction, which are also core contributors to the progression of CHF^[50,51]^, The dysregulation of the RAAS Activated by low renal perfusion or increased sympathetic activity^[52]^, RAAS leads to the release of renin, which converts angiotensinogen to angiotensin I, and ACE further converts this to angiotensin II. Angiotensin II triggers vasoconstriction, both directly and through the release of vasopressin and norepinephrine, increasing systemic vascular resistance^[53]^. It also stimulates renal sodium retention and aldosterone release, further exacerbating volume retention. This cycle of vasoconstriction and volume overload paradoxically worsens the hemodynamics of ADHF^[41,54]^. Other vasoconstrictor pathways, such as endothelin-1, are also activated, contributing to peripheral vascular constriction and further elevating blood pressure^[41]^. Along with vasopressin release, it leads to sodium and water retention and vasoconstriction, which increase ventricular preload and afterload, elevating wall stress. This triggers the production of pre-pro B-type natriuretic peptide (BNP), which is cleaved into active BNP and inactive NT-proBNP^[55]^. BNP promotes natriuresis and vasodilation to reduce the detrimental effects of fluid overload^[56]^. Similarly, atrial stretch produces atrial natriuretic peptide (ANP) with similar effects, and urodilatin, a peptide related to ANP, is produced in the kidneys. C-type natriuretic peptide is another member of the system, released from endothelial cells, but is found in lower concentrations in the blood, and also supports vasodilation and diuresis through similar pathways^[57]^. Despite these compensatory mechanisms, the inactivation of natriuretic peptides by neprilysin limits their effectiveness^[58]^. Regarding neprilysin in this context, neprilysin is a circulating enzyme responsible for breaking down natriuretic peptides like BNP and ANP, as well as other beneficial peptides such as bradykinin. In HF, inhibiting neprilysin can help sustain the positive effects of these peptides, which counteract fluid retention, vasoconstriction, and other detrimental effects of ADHF (Fig. 2)^[59]^.

#### Endothelial activation

One other important pathophysiological component of ADHF is endothelial dysfunction, which is triggered by both neurohormonal activation and biomechanical stress. In response to hydrostatic pressure and shear stress, the endothelium triggers mechanosensors that increase the synthesis of nitric oxide (NO) and reactive oxygen species (ROS)^[60]^. Endothelial nitric oxide synthase produces NO, which normally promotes vasodilation and anti-inflammatory effects^[61]^. However, too much ROS lowers the bioavailability of NO, which results in oxidative stress, vasoconstriction, and compromised vascular compliance^[62]^. By inhibiting NO production, encouraging ROS creation, and starting inflammatory signaling via the NF-κB pathway, neurohormonal hormones such as angiotensin II (ATII), aldosterone, norepinephrine, and vasopressin further exacerbate endothelial dysfunction^[63,64]^. ATII promotes leukocyte infiltration and inflammation by upregulating adhesion molecules (ICAM-1 and VCAM-1) and inflammatory mediators such as IL-6, TNF-α, and MCP-1^[65]^. Additionally, aldosterone promotes collagen synthesis, smooth muscle cell proliferation, and intima-media thickening, all of which increase arterial stiffness and contribute to vascular remodeling. Nitrotyrosine and COX-2, two indicators of endothelial oxidative stress and inflammation^[66]^, are increased in ADHF patients; these levels have been shown to decrease after clinical stabilization. These mechanisms highlight how endothelial dysfunction plays a dynamic role in driving vascular impairment and unfavorable outcomes in ADHF (Fig. 2)^[67]^.

### Hypervolemic vs. hypovolemic ADHF

Differentiating between hypervolemic and hypovolemic ADHF is crucial for effective management^[68]^. Hypervolemic ADHF is characterized by fluid overload. In contrast, hypovolemic ADHF, or cardiogenic shock, involves inadequate tissue perfusion. Accurately identifying the underlying hemodynamic profile ensures targeted therapy, improving patient outcomes^[69]^. It is a complex clinical state that results from structural or functional abnormalities of the heart, which impede the heart’s ability to fill with and/or eject blood into the circulation, leading to excessive accumulation of fluid within the body (Table 1)^[70]^. Structural or functional heart abnormalities reduce cardiac output and increase intraventricular pressure, affecting exercise tolerance^[71]^. Myocyte regeneration, myocardial hypertrophy, and Frank-Starling mechanism are compensatory mechanisms that sustain the cardiac output initially but eventually result in fibrosis and reduced functioning of the heart^[72]^. The elevated pulmonary capillary pressures combined with inflammatory lung injury account for the pulmonary congestion hallmark of CHF, causing fluid to accumulate in the alveoli and giving rise to dyspnea^[73]^. Furthermore, hypoalbuminemia contributes to pulmonary congestion, as the colloid osmotic pressure is too low to keep the fluid inside the vessels and outside the lung tissues (Table 1)^[73]^.

**Table 1:** Comparison of hypervolemic and hypovolemic ADHF

Aspect	Hypervolemic ADHF	Hypovolemic ADHF
Primary mechanism	Fluid overload	Decrease cardiac output
Clinical presentation	Congestion, edema, dyspnea	Hypoperfusion, fatigue, hypotension
Treatment focus	Diuretics, vasodilators	Fluid resuscitation, vasopressors

On the other hand, hypovolemic shock results from such a considerable amount of intravascular volume loss that leads directly to decreased preload, stroke volume, and cardiac output^[74]^. Precisely the opposite, this is compared to a hypervolemic state, generally due to fluid overload, which initiates mechanisms to compensate for the increased systemic vascular resistance to maintain perfusion^[75]^. This leads to tissue hypoperfusion, failure of organs, and eventual death if not timely intervened. The most frequent causes include acute hemorrhage, excessive fluid loss through vomiting or diarrhea, and third-spacing, where fluid shifts into nonvascular compartments which has been shown in burns or postoperative states^[76]^. Unlike the gradual fluid accumulation that occurs in hypervolemic conditions, hypovolemic shock will result from rapid depletions, thus demanding immediate fluid resuscitation^[77]^. The coexistence of the so-called “lethal triad” of acidosis, hypothermia, and coagulopathy will probably make the condition worse, especially in cases of uncontrolled hemorrhage. Volume replacement and bleeding control must be performed as quickly as possible to prevent lethal outcomes (Table 1)^[68]^.

### Etiologies of acute decompensated HF

It is these multifaceted causes of ADHF events that help shape their clinical course, management strategies, and outcomes. Most of these causes can commonly be identified: ischemic heart disease, hypertensive heart disease, and valvular causes are the most common causal agents, especially in elderly patients^[78]^. Rheumatic heart disease and cardiomyopathies such as Chagas cardiomyopathy play a major role in the burden of ADHF in the economically deprived parts of the world^[79]^. The most striking features of Chagas disease are cardiogenic shock, which accounts for 15%; arrhythmias, amounting to 20.4%; and right ventricular (RV) dysfunction with ascites in 27.4%, hepatomegaly in 48.7%, and jugular distension in 61.9%. Patients with Chagas cardiomyopathy have a worse prognosis compared to ischemic and hypertensive etiology mechanisms, given mechanisms such as autonomic dysfunction, ventricular arrhythmias, and thromboembolic events^[80]^.

Hypervolemic and hypovolemic states represent two sides of the same coin, but are important complementary aspects in the etiology of ADHF^[81]^. Hypervolemic ADHF could be a consequence of systemic inflammation, increased pulmonary capillary pressures, and fluid retention, which is often associated with conditions such as ischemic heart disease and chronic hypertension^[48]^. The mechanisms presented in this context consequently cause congestion, increased preload, and multiorgan dysfunction^[82]^. On the other hand, hypovolemic shock occurs as a result of severe intravascular fluid loss that reduces preload, stroke volume, and consequently cardiac output^[83]^. It is classically caused by acute bleeding, severe diarrhea, vomiting, and/or third-space loss of fluids in burns or postoperative settings. Whereas hypervolemia primarily leads to congestion and organ edema, hypovolemia leads to tissue hypoperfusion, rapid organ failure, and death if not treated^[3]^. Being aware of these diverse etiologies of ADHF is very important in directing treatment and prognosis. This understanding now shifts the importance toward examining the clinical presentation and manifestations that result from these etiologies.

### Clinical presentation and diagnostic considerations

Among the patients who visited the emergency department, dyspnea presented as the most common symptom of acutely decompensated HF in 73.1% of the patients^[84]^. Other frequent complaints include orthopnea, lower limb edema, which was reported in the cases, exercise intolerance, palpitations, presyncope, and abdominal bloating, mainly due to increased intravascular congestion in nature^[85]^. In particular, abdominal bloating is due to the increase in fluid of the splanchnic circulation, while hepatic and gastrointestinal edema further increase abdominal distension and systemic congestion^[86]^. At enrolment, LVEF was reduced in 49.3% of hospitalized patients and 59.5% of outpatients, while an overweight condition was present in 38.6% of hospitalized patients and 41% of outpatients^[87]^. At admission, the levels of median creatinine and blood urea nitrogen were raised in hospitalized patients, 1.2 and 32.0 mg/dL, respectively, compared to 1.1 and 26.0 mg/dL in outpatients^[88]^. Symptoms include fatigue, exercise intolerance, peripheral edema, and increased jugular venous pressure as manifestations of systemic congestion and poor organ perfusion. Examination typically discloses pulmonary crackles, pleural effusions, an S3 gallop, and murmurs of mitral or tricuspid regurgitation^[89]^. This, in turn, raises RV pressures further by causing pulmonary congestion. The result of this is a downward spiral with multi-organ dysfunction. Neurohormonal compensatory mechanisms, frequently manifesting as increased chronotropy and inotropy, will often result in tachycardia, arrhythmias, and myocardial strain^[90]^. Right HF contributes further to peripheral congestion and end-organ damage, usually secondary to left HF but sometimes in isolation from it. Also, levels of natriuretic peptide, which come from neurohormonal mechanisms, it’s a crucial sign of myocardial stretch, and vary greatly in patients with ADHF^[91]^. Those patients who have low levels of NP at the time of admission are usually seen to have lower PCWP and can be able to be discharged in shorter lengths of stay, because their myocardial wall stretch had not become great enough to trigger the release of NPs^[92]^.

Features of ADHF, very high levels of PCWP and low CI, highlight the great importance of meticulous diagnosis and attention to specific interventions to stabilize hemodynamics and prevent further complications^[93]^. These findings, derived from the AMERICCAASS Registry, emphasize the interplay between ventricular dysfunction, vascular tone, and neurohormonal dysregulation in the progression of ADHF^[94]^.

## Current guidelines for management of acute exacerbation

Having discussed the different types of acute HF exacerbations, the focus shall shift to elaboration of their management as it stands in clinical practice. Current guidelines have an important place in this aspect, with clear, evidence-based recommendations that help clinicians make timely and effective decisions. Guidelines are constructed to address patient needs precisely in an acute episode of the disease, guiding immediate treatment and longer-term strategies for care aimed at overall outcome improvement.

### Overview of key guidelines

**Figure 3.**
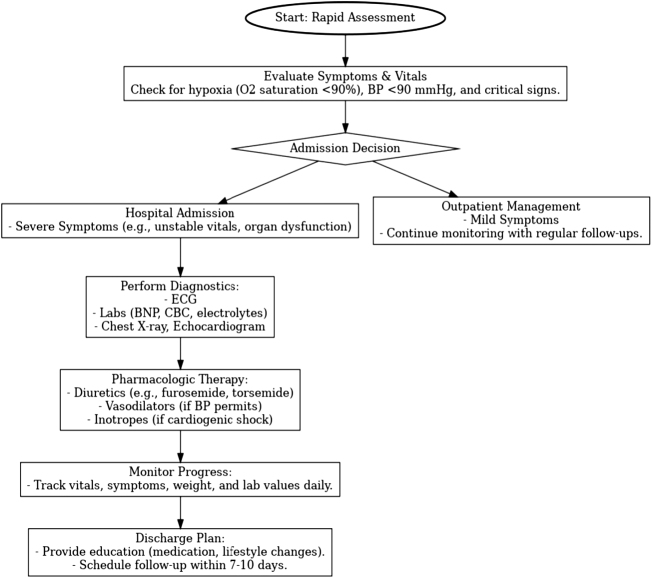
Flowchart for rapid assessment and management of heart failure. This flowchart outlines the step-by-step process for the rapid assessment and management of heart failure. Starting with symptom evaluation and vital assessment, it guides decision-making based on the severity of symptoms. Patients with severe symptoms are directed toward hospital admission, diagnostics, pharmacologic therapy, and monitoring, followed by a structured discharge plan. Those with mild symptoms are managed as outpatients with regular follow-ups.

The management of the acute HF exacerbation follows key recommendations by some organizations, such as ACC, AHA, and ESC^[95,96]^. These are guidelines that recommend a systematic approach to the identification and treatment of exacerbations: rapid assessment, relief of symptoms, and stabilization. They enumerate some specific pharmacologic therapies to improve symptoms and optimize cardiac output, including diuretics, vasodilators, and inotropes^[95]^. Recent updates from ACC, AHA, and ESC have centered around the management strategies of peripheral artery disease and its associated disorders, predominantly in patients with diabetes or at very high cardiovascular risk^[97,98]^. The 2024 ACC/AHA guidelines recommend a combination of low-dose rivaroxaban plus aspirin to reduce major cardiovascular events and limb events in symptomatic PAD patients (Fig. 3)^[97]^. LDL-C goals have also been lowered further to <55 mg/dL, and intensive statin therapy was emphasized first, followed by the addition of PCSK9 inhibitors or ezetimibe^[97]^. For those patients with PAD and type 2 diabetes, GLP-1 receptor agonists and SGLT2 inhibitors are also recommended for further reduction in cardiovascular risk. Systolic blood pressure targets have now been tightened to 120–129 mmHg in patients with PAD, reflecting the trend toward aggressive risk management^[98]^. The statements regarding hospital admission in the guideline reinforce symptom severity, hemodynamic instability, and comorbid conditions, providing criteria for safe discharge to home, with individualized plans necessary for reducing readmission risk (Fig. 3)^[95,96]^.

An intense, step-by-step method for the management of an admitted patient with HF involves evaluation and treatment. The first step in assessing a patient is the determination of his symptoms, vital signs, and hemodynamic stability-blood pressure, heart rate, respiratory rate, and oxygen saturation-through physical examination to identify the severity of the exacerbation and determine if the patient requires hospital admission^[99]^. So we need to obtain a complete clinical history by asking the patient about dyspnea, cough, nocturia, and generalized fatigue, The late stage of HF may cause gastrointestinal symptoms, such as, abdominal bloating, anorexia, and fullness in the upper right quadrant (Fig. 3)^[100]^. It is very important to ask about hypertension, since blood pressure is the second most frequent cause of HF^[99]^. Indications for hospitalization include severe symptoms, significant congestion, new-onset HF, and high medical risk; however, it is important to avoid unnecessary admissions to decrease risks such as nosocomial infections^[97]^. Key diagnostic tests at admission will also include ECG in the diagnosis of arrhythmias or ischemia, chest X-ray to identify pulmonary congestion, echocardiogram to assess left ventricular function, and laboratory blood tests like CBC, metabolic panels, and biomarkers such as BNP or NT-proBNP for the assessment of cardiac status, kidney function, and other systemic conditions (Fig. 3)^[100,101]^.

Once admitted, the patient’s progress will need to be closely monitored, including frequent vital signs initially (q 4–6 hours), weights daily, and fluid status regarding congestion. Dyspnea, fatigue, and peripheral edema should also be followed daily in the assessment of response to measure and adjust therapy^[98]^. Diuretic effect shall be monitored by weight change, urine output, and symptoms of congestion, making dose adjustments according to the patient’s response^[97]^. Repeat key laboratory tests, such as electrolytes, renal function, and BNP, which may indicate changes due to either therapy or the development of complications in those receiving high-dose diuretics or vasoactive agents. Consider dose adjustments based on findings, refer for cardiology consultation in cases of persistent symptoms despite standard therapy, or advanced therapies, such as inotropes or mechanical support. Such an approach guarantees comprehensive care that integrates detailed initial assessments with regular monitoring and adjustments of therapies to tailor them to each patient’s ever-changing condition (Fig. 3)^[97,98]^.

Thresholds in hospital admission or outpatient management in HF include those outlined by the HFSA regarding the determination to admit a patient to the hospital or manage him/her as an outpatient^[102]^. The patients who, despite maximization of outpatient therapy, experience frequent exacerbations usually require admission to the hospital, as frequent exacerbations are indicative of a need for closer monitoring, and perhaps more aggressive modification of therapy^[103]^. A systolic blood pressure less than 90 mmHg is another critical threshold, as it is indicative of low perfusion and hence indicative of hemodynamic instability that may require intravenous medications or close hemodynamic monitoring possible only in an inpatient setting. Decreased renal function alone, particularly with rapid changes in creatinine or significant electrolyte disturbances, also calls for hospital admission. These cases complicate not only volume management but also limit the safe use of certain HF medications; thus, hospitalization is necessary to allow safe adjustments in therapy. Other high-risk features favoring admission include severe congestion unresponsive to outpatient diuretics, new or progressive symptoms, or the comorbid presence of severe arrhythmias, recent myocardial infarction, or worsening pulmonary or liver function. This would ensure that a high-risk HF gets the benefit of intensive monitoring and treatment to improve outcome and prevent further deterioration (Fig. 3)^[96]^.

### Pharmacologic therapies in acute HF exacerbation

Loop diuretics are the first-line therapy for decongestion in acute HF and consist of intravenous furosemide (20–40 mg) or torsemide (10–20 mg). The early response is optimally quantified by measuring urine output, with a target of 100–150 ml per hour during the early phase. Subsequent increases in diuretic doses should be doubled if urine output remains below this threshold until maximal levels are achieved. Low urine output of less than 100 ml/hr that is persistent despite dose escalation reflects an inadequate response, and combination diuretic therapy can be considered, including adding hydrochlorothiazide or metolazone. Combination therapy may enhance the diuretic effect, but at increased risk for hypokalemia and impaired kidney function; therefore, frequent monitoring of electrolytes and renal function is important to minimize complications (Table 2)^[104]^.

**Table 2:** Therapeutic roles, indications, and considerations for key drugs in acute heart failure management

Drugs	Therapeutic role	Indications	Key considerations	Reference
Loop diuretics	Initial diuretic therapy	First-line for decongestion in acute heart failure	Includes **furosemide** (20–40 mg) or **torsemide** (10–20 mg); target urine output of 100–150 ml/hr; monitor electrolytes and kidney function due to risk of hypokalemia.	[104]
Combinations diuretics	Enhanced diuretic effect	Used when loop diuretics alone are insufficient	Add **hydrochlorothiazide** or **metolazone** for enhanced effect; increased risk of electrolyte disturbances, requires close monitoring.	[104]
Empagliflozin	Adjunctive therapy	Early adjunct for enhanced decongestion	SGLT2 inhibitor, improves urine output without compromising kidney function; not to be used with **acetazolamide** due to limited interaction data.	[105]
Acetazolamide	Adjunctive therapy	Early adjunct for enhanced decongestion	Administered intravenously (500 mg daily for 3 days); watch for effects on potassium and blood pressure; not recommended with SGLT2 inhibitors.	[105]
Vasodilators	Reduced preload and afterload	Indicated in hypertension or ischemia complicating acute heart failure	Avoid in patients with systolic blood pressure <90 mmHg; monitor blood pressure and kidney function, especially in renal impairment.	[106]
Anticoagulants	Thrombosis prevention	High-risk patients (reduced mobility or thromboembolic risk)	Options include **heparin**, **LMWH**, **warfarin**, or **DOACs**; choice depends on individual patient risk factors and renal function.	[106]
Inotropes	Increase cardiac output	Cardiogenic shock with low systolic BP (<90 mmHg)	Includes **dobutamine**, **dopamine**, **milrinone**, and **levosimendan**; use in critical care settings only, with continuous ECG monitoring to manage arrhythmia risks.	[107]

Also, adding 25 mg of empagliflozin daily within the first 12 hours of admission has shown a 25% increase in urine output without compromise in kidney function and may be complementary to loop diuretics to enhance decongestion^[105]^. Early intravenous acetazolamide, 500 mg daily for 3 days, similarly improves decongestion rates by day three without significant effects on potassium levels, blood pressure, or renal function^[108]^. However, for the time being, empagliflozin and other SGLT2 inhibitors should not be used with acetazolamide, as their interaction has been minimally reported (Table 2)^[109]^.

Furthermore, Vasodilators are indicated in patients with hypertension or ischemia complicating acute HF, having the benefit of decreasing both preload and afterload. Their use is contraindicated in those with low systolic blood pressure (<90 mmHg) because of the risk of further hypotension and perfusion^[110]^. In addition, therapy with vasodilators needs adjustment both in borderline blood pressure and in renal impairment, as aggressive vasodilation may be associated with worsening renal function. This, therefore, is going to require close monitoring of blood pressure and renal parameters to maintain hemodynamic stability during treatment. Anticoagulation for hospitalized patients with HFrEF has been recommended to prevent deep vein thrombosis (Table 2). This includes either heparin, LMWH, warfarin, or DOACs, especially in patients with reduced mobility or other thromboembolic risk factors. The selection of these anticoagulants depends on individual risk factors and renal function^[106]^.

As for the routine use of opiates for the management of acute HF it is best avoided, since it has been associated with poor outcomes related to increased rates of mechanical ventilation, admissions to the ICU, lengths of hospital stay, and mortality. Opiates should generally be avoided except when necessary because their administration can worsen both respiratory and hemodynamic parameters^[104]^.

Inotropes are mainly indicated for patients in cardiogenic shock with low cardiac output and systolic blood pressure less than 90 mmHg^[111]^. Agents like dobutamine, dopamine, milrinone, and levosimendan could improve cardiac output and tissue perfusion, but also carry risks of inducing arrhythmias and increasing oxygen demand. Inotropes should be used in a critical care setting with continuous ECG and hemodynamic monitoring to identify and manage adverse effects promptly. The routine use of inotropes in hemodynamically stable patients is not recommended, given the associated risks (Table 2)^[107]^.

While beta-blockers are responsible for reducing long-term mortality in the chronic management of HF by blunting the deleterious effects of sympathetic activation. They are indicated for initiation after stabilization, post-acute phase, to offer continued cardio protection, and to slow the progression of the disease^[112]^.

Finally, in acute heart failure exacerbations (AHFE), oxygen therapy is essential for patients with hypoxemia (oxygen saturation <90%) to alleviate tissue hypoxia and improve clinical outcomes. However, its use in non-hypoxemic patients can be detrimental, leading to increased systemic vascular resistance, reduced cardiac output, and worsened prognosis. Current recommendations suggest avoiding routine oxygen supplementation in AHFE patients with normal oxygen saturation, as it offers no benefit and may cause harm. These findings highlight the need for careful assessment and tailored oxygen use based on individual oxygenation status^[113]^.

### Discharge plan

The discharge planning for patients with HF should be done very meticulously by assessment of clinical stability along with a coordinated approach for continuity of care after hospital discharge^[114]^. That is, the patient should have the following criteria when discharged: NYHA class I or II that is stable for 6–12 months; on optimal pharmacological therapy; the patient has stable adherence; no recent hospital admissions for more than 1 year; and an LVEF >35%^[115]^. The monitoring before discharge would have been verification of stable vital signs, including blood pressure and heart rate, and resolution or management of symptoms such as edema, dyspnea, and fatigue. Other key parameters, including urine output, kidney function, and response to diuretics, must also be taken into consideration. Once stability is achieved, multidisciplinary teams of physicians, nurses, pharmacists, and social workers should combine efforts in educating the patient and caregivers about lifestyle changes: limiting sodium and fluids, monitoring weight, and exercising. The team will also facilitate medication adherence, address potential barriers to home care, and schedule follow-up appointments with primary care providers and HF specialists within 7–10 days^[116]^. This could include remote monitoring or a visit to a HF clinic if appropriate^[96]^. The patient should be well-educated about the signs of HF exacerbation and the emergency protocol. With these in place, the discharge plan aims at clear instructions, easy follow-up, and reduced chance of readmission^[117]^.

## Role of beta-blockers in acute HF exacerbation

**Figure 4.**
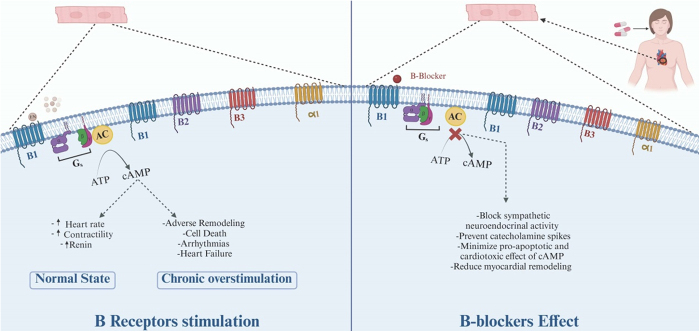
Mechanism of action of beta-blockers in cardiac function. The figure illustrates the mechanism of beta-blockers on β1 and β2 ARs. The left panel shows normal stimulation by catecholamines, increasing cAMP, heart rate, and contractility, but chronic overstimulation leads to adverse cardiac effects. The right panel demonstrates beta-blockers inhibiting receptor activation, reducing cAMP, sympathetic activity, and myocardial remodeling, providing therapeutic benefits in cardiac conditions.

The human heart has different types of adrenergic receptors (ARs), with the prominent ones being β1, β2, and α1. In individuals without HF, 80% of the ARs are β2. However, in individuals with HF, β2 receptors are downregulated, resulting in an overall ratio of ARs of 2:1:1 for β1, β2, and α1, respectively^[118]^. β-adrenergic receptors (β-ARs) are coupled to adenylyl cyclase through the stimulatory G protein Gs, and they have a positive inotropic effect on the myocardium (Fig. 4)^[119]^. As end-organ perfusion is altered in HF, multiple neuroendocrine mechanisms (e.g. activation of the RAAS and the SNS) are triggered to help maintain perfusion. In 1975, a study by Waagstein *et al*^[120]^ suggested that increased catecholamine activity can be an important component in the pathophysiology of HF. Chronic overstimulation in HF patients negatively affects cardiac myocytes’ function^[121]^. Sustained β-ARs stimulation leads to cell death, adverse remodeling, and arrhythmias^[122]^.

There are multiple generations of β-blockers. First-generation β-blockers are non-selective, working equally on both β1 and β2 receptors. Propranolol and timolol are common examples of this generation. Second-generation β-blockers are cardioselective, acting primarily on β1 receptors with much less effect on β2 receptors. Metoprolol and bisoprolol are well-known examples of this generation. Third-generation β-blockers, such as carvedilol, work on both β1 and β2 receptors while also blocking α1 receptors. Additionally, carvedilol possesses antioxidant properties.

The usage of β-blockers for chronic HF has undergone a long journey, evolving from being considered a hazardous drug to becoming one of the most effective treatments for reducing mortality in patients with chronic HF. The positive effects of β-blockers for patients with HF were first suggested in 1975 by Waagstein *et al*^[120]^ Nowadays, it is well-established that early β-AR blockade is essential for patients with HFrEF^[122,123]^. This effect can be mainly explained by the β-blocker’s ability to block sympathetic neuroendocrine activity, prevent catecholamine spikes, lower heart rate, and minimize the pro-apoptotic and cardiotoxic effects caused by calcium overload from cyclic adenosine monophosphate. They are among the few drugs shown to reduce mortality in patients with HF (Fig. 4)^[118,121,124]^.

Chronic HF was initially thought to be purely a hemodynamic disease. However, further studies have revealed that the disease process also involves cardiac remodeling and inflammation driven by neuroendocrine activity, which β-blockers effectively prevent^[125,126]^. Conversely, the role of β-blockers in acute decompensated or acute exacerbation of HF remains controversial^[122]^.

In acute settings, that is, acute exacerbation of HF, hemodynamic alterations are the primary contributors to symptoms. While inotropic agents are generally not used in the management of chronic HF, they may play a role in cases of acute exacerbation. For patients with chronic HF who are on β-blockers, acute cessation of β-blockers can have a positive inotropic effect^[123]^.

Inotropic agents may be beneficial in severe cases of acute exacerbation of HF with systolic impairment, as they can enhance perfusion to end organs. However, their use can be harmful in milder cases due to the increased myocardial oxygen demand caused by heightened inotropy of cardiac myocytes (Table 3)^[123]^. According to the ACCF/AHA guidelines, inotropic agents are recommended in patients with cardiogenic shock as a temporary measure to maintain perfusion to vital organs until definitive treatment can be performed. However, their use can lead to cardiac myocyte damage, potentially worsening the baseline HF after the acute episode resolves (Fig. 4)^[127]^.

**Table 3:** Impact of beta-blocker use on the efficacy of inotropic agents in AHF

Inotropic agent	Mechanism of action	Impact of beta-blocker (BB) use	Key findings	Potential benefit in AHF with BB
Dobutamine	β1 and β2 agonist (catecholamine)	Effects are reduced, especially with carvedilol^[122]^	Requires beta-receptor stimulation, so BB, particularly carvedilol, attenuates hemodynamic effects^[122]^	Requires higher doses with BB^[122]^
Milrinone	PDE inhibitor; increases CO by lowering SVR and LVEDP^[122]^	-	Randomized trials show similar hemodynamic effects to dobutamine but no survival benefit in DOREMI study^[122]^	No proven mortality benefit^[128]^
Enoximone	PDE inhibitor; increases CO, and lowers SVR^[122]^	Not affected by BB (even with carvedilol)^[129]^	PDE inhibition may stabilize hemodynamics in BB-treated patients, especially those on carvedilol^[129]^	-
Levosimendan	Calcium sensitizer; increases CO, lowers SVR	Effects not impacted by BB^[130]^	SURVIVE Study suggests reduced all-cause mortality in BB-treated patients with AHF compared to dobutamine^[131]^	Shown to reduce mortality in AHF patients with BB^[131]^

Theoretically, continuing β-blockers during acute exacerbation could worsen the condition, as the inotropic status of the heart is critically important in ADHF with reduced systolic function^[132]^. However, a randomized controlled study by Prins *et al*^[133]^. Concluded that discontinuation of β-blockers increases in-hospital mortality and leads to poorer outcomes (Table 4). These findings suggest that continuing β-blockers in these patients is advisable if the clinical picture allows (Table 5).

**Table 4:** Critical evaluation of cited clinical trials

Trial name	Type and phase	Population	Quality assessment	Key limitations	Reference
GALACTIC-HF	RCT, phase III	Chronic HFrEF patients	Large-scale, high-quality, well-powered	Post hoc analyses limit generalizability	Teerlink *et al*, 2021
DREAM-HF	Phase III	Patients with chronic HF	Strong biologic rationale, flexible design	No final results reported	Butler *et al*, 2019
Splanchnic-HF-2	Pilot RCT	15 chronic HF patients	Innovative device-based strategy	Very small sample size	Abraham *et al*, 2020
RED DESERT & SAHARA	Device trials	HF patients (euvolemic/hypervolemic)	First-in-human, biomarker follow-up	Non-randomized, small samples	Costanzo *et al*, 2021
TARGET-1 & TARGET-2	Device trials	ADHF patients	Promising safety/efficacy data	Non-randomized, low N	Grodin *et al*, 2021
CPNS First-in-Human	Feasibility study	7 HF patients	Novel mechanism, invasive monitoring	Tiny sample, early-stage	Neuzil *et al*, 2022
ADVOR Trial	RCT	Acute HF patients with volume overload	Strong evidence base, peer-reviewed	Electrolyte risks, renal impact	Mullens *et al*, 2022
FAIR-HF & AFFIRM-AHF	RCTs	Chronic HF with iron deficiency	High-quality, large-scale studies	Application in acute HF is less clear	Anker *et al*, 2009; Ponikowski *et al*, 2020
STRONG-HF	RCT	Acute HF patients	Multinational, randomized	Open-label design may bias results	Mebazaa *et al*, 2022
TRANSITION	RCT	Stabilized hospitalized HF patients	Real-world, practical outcomes	Early-phase hospitalization only	Wachter *et al*, 2019
PIONEER-HF	RCT	Hospitalized HFrEF patients	Strong clinical endpoint focus	Short follow-up duration	Velazquez *et al*, 2019
B-CONVINCED, OPTIMIZE-HF	Observational & RCT	Acute HF on beta-blockers	Supports beta-blocker continuation	Subgroup variation, real-world confounding	Fonarow *et al*, 2007; Nieminen *et al*, 2006
ESCAPE, CARRESS-HF, UNLOAD	RCTs	Severe HF, congestion, monitoring	Foundational trials, mixed outcomes	Mixed results and selective use issues	Costanzo *et al*, 2007
SOLOIST-WHF	RCT	HF + T2D	Early SGLT2 efficacy data	Premature termination affects power	Bhatt *et al*, 2021
TRANSFORM-HF	Pragmatic RCT	Comparing diuretics in HF	Practical, head-to-head	Outcomes pending	Mentz *et al*, 2023
REVERSE-HF	RNA-therapy trial	Molecular-targeted HF patients	Cutting-edge genomic design	Still under investigation	Zannad *et al*, 2023

**Table 5:** Key studies on the role of beta-blockers in heart failure management and outcomes

Study	Population/focus	Key findings
Prins *et al*^[133]^	Patients with acute decompensated heart failure (ADHF).	Continuation of β-blockers is significantly associated with reductions in the risk of in-hospital mortality, short-term mortality, and the combined endpoint of short-term rehospitalization or death.
Ouwerkerk *et al*^[134]^	Chronic HF patients receiving ACE inhibitors and β-blockers.	Higher β-blocker and ARBs/ACE-inhibitor doses reduced death and hospitalization risks.
Metra *et al*^[135]^	Post-HF hospitalization β-blocker adjustments.	Dose reduction or cessation increased death risk.
Tamaki *et al*^[136]^	Use of β-blockers at admission for acute HF.	Use of β-blockers at admission is associated with lower in-hospital mortality, regardless of LVEF or ischemic etiology.

Holding β-blockers may not be effective in acute settings for several reasons, including the following: first, delayed drug clearance, in which abrupt cessation of β-blockers in patients with chronic use does not mean immediate effects, as these drugs require time to clear from the body. For example, bisoprolol has a half-life of approximately 11 hours, and carvedilol has a half-life of 6–10 hours. Second, suppose β-blockers were recently introduced or their dosage was recently adjusted. In that case, it may be more appropriate to focus on treating the underlying precipitating cause of the acute episode rather than discontinuing the medication^[123]^. Third, abrupt discontinuation of β-blockers can lead to a rebound adrenergic effect, potentially inducing angina and significantly increasing the risk of sudden death in this patient population (Table 3)^[137–139]^.

The approach to determining whether to stop, reduce, or continue β-blockers in patients with AHF depends on their hemodynamic status and whether they are already on β-blockers. Patients with AHF can present as hemodynamically stable, hypoperfused (defined as a CI <2.2 L/min with evidence of volume overload, lactate >2 mmol/L, or urine output <30 mL/h), or in cardiogenic shock^[122]^.

In patients with acute heart failure (AHF) who were not on β-blockers before the onset of the episode and are hemodynamically stable with no signs of hypoperfusion, the initiation of β-blockers before discharge is essential, provided that hemodynamic stability is maintained. As concluded by Ouwerkerk *et al*^[134]^, patients receiving 100% of the recommended doses of β-blockers and ACE inhibitors/ARBs had a lower risk of death and/or HF compared to those receiving suboptimal doses (Table 3).

In patients with ADHF who were already on β-blockers before the onset of the episode and are hemodynamically stable with no signs of hypoperfusion, studies support the continuation of β-blockers without dose reduction. A randomized controlled study by Prins *et al*^[133]^ concluded that discontinuing β-blockers increased in-hospital mortality, short-term mortality, and the combined endpoint of short-term rehospitalization or mortality. The study suggests several mechanisms to explain these effects, including that Patients started on β-blockers before discharge are more likely to adhere to therapy 60 days post-discharge compared to those who were not (91.2% vs. 73.4%)^[140]^. Furthermore, β-blockers reduce arrhythmias, particularly in cases where inotropic agents, known to have pro-arrhythmic properties, are used. These findings suggest that continuing β-blockers in these patients is advisable if the clinical picture allows (Table 3).

Metra *et al*^[135]^ examined the relationship between changes in β-blocker doses in HF patients following an episode of HF hospitalization in the COMET study (Carvedilol or Metoprolol European Trial). Their results demonstrated that both reduction and cessation of β-blockers were associated with an increased risk of death. These studies underscore the detrimental effects of reducing or discontinuing β-blockers in patients with ADHF who were already on β-blockers and showed no signs of hypoperfusion (Fig. 4).

Schurtz *et al*^[122]^ suggest that reducing or discontinuing β-blockers in patients with no signs of hypoperfusion should be reserved for high-risk patients. These include individuals with refractory congestion, severe bradycardia, hemodynamic instability without hypoperfusion, severe RV failure, or severe renal impairment (Table 3).

Managing ADHF patients with preexisting β-blocker therapy who show signs of hypoperfusion requiring the use of inotropic agents is particularly challenging. The table below summarizes different inotropic agents, their mechanisms of action, the impact of β-blocker (BB) use, key findings, and their potential benefits in ADHF patients on BB therapy.

Deciding whether to completely discontinue, adjust the dose, or continue β-blockers in ADHF with hypoperfusion is highly challenging. Evidence supporting any approach is limited; however, some experts have combined their clinical expertise with available studies to make the following recommendations, as outlined by Schurtz *et al* β-blockers should be discontinued in patients with cardiogenic shock classified as stage C–E of the Society of Cardiovascular Angiography and Intervention (SCAI) classification^[128]^. β-blockers should not be stopped in patients who are hemodynamically unstable but show no signs of hypoperfusion (SCAI shock stage B, also referred to as beginning cardiogenic shock/compensated shock/pre-shock).

**Figure 5.**
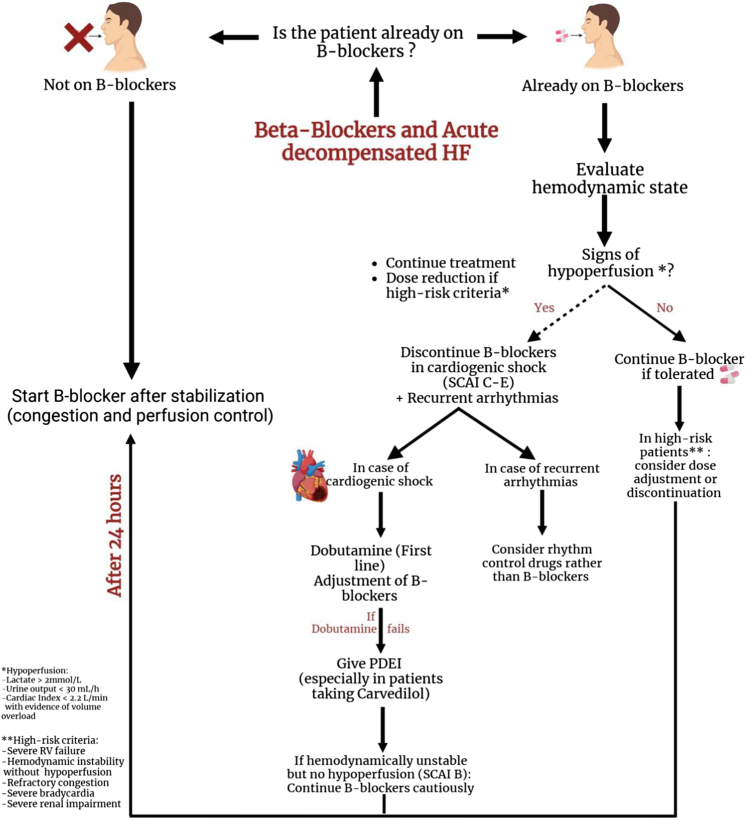
Management of beta-blockers in ADHF. This flowchart outlines the decision-making process for managing beta-blocker therapy in patients with ADHF. The pathway distinguishes between patients already on beta-blockers and those who are not, considering their hemodynamic state. Key steps include assessing signs of hypoperfusion, such as elevated lactate levels, reduced urine output, low cardiac index, and volume overload. Management options include continuing, adjusting, or discontinuing beta-blockers based on clinical presentation, with specific interventions for cardiogenic shock and recurrent arrhythmias. In cases of hemodynamic instability without hypoperfusion, beta-blockers may be continued cautiously. Initiation of beta-blockers in naïve patients is recommended only after stabilization, typically 24 hours after achieving control of congestion and perfusion.

In cases of cardiogenic shock triggered by recurrent ventricular arrhythmias, other rhythm-control drugs may be preferred over β-blockers. While the inotropic effect of dobutamine may require higher doses in the presence of β-blockers^[129]^ (Fig. 5), it is still considered preferable to other inotropic agents, particularly for patients not previously on β-blockers or those who recently started β-blocker therapy. Phosphodiesterase (PDE) inhibitors may be considered if dobutamine fails, especially in patients taking Carvedilol. Reintroducing or initiating β-blockers in patients with AHF should be done as soon as clinical stabilization is achieved (i.e. congestion and perfusion are controlled) and vasoactive agents are weaned, typically after 24 hours (Table 5).

Tamaki *et al*^[130]^ found that, regardless of left ventricular ejection fraction or ischemic etiology, the use of β-blockers at admission is significantly associated with a lower risk of in-hospital death. This study postulates three possible mechanisms that may explain this favorable outcome: β-blockers suppress the SNS, as evidenced by a lower heart rate, reduced preload, and decreased afterload (Fig. 5). In ADHF, increases in any of these parameters can worsen the condition and place additional stress on the heart; reduce AF risk; and finally, reduce the risk of sudden cardiac death, particularly in patients with a history of arrhythmias or previous hospitalizations for such episodes (Table 5).

Further support for these findings comes from Jacob *et al*^[131]^, whose study showed that patients chronically treated with β-blockers had better short-term outcomes compared to those who were not. However, a study conducted by Bok *et al*^[141]^, compared the outcomes of patients with ADHF who were on β-blockers for CHF. The study focused on two groups: patients whose β-blocker doses were adjusted (discontinued or reduced) and those whose doses were maintained as prescribed before the acute decompensation episode. The primary outcomes included arrhythmias, such as non-sustained or sustained ventricular tachycardia, ventricular fibrillation, or AF/flutter with rapid ventricular response during hospitalization. The study found no significant difference in outcomes between the two groups (Fig. 5).

Upon discharge, for patients who survived an episode of ADHF, Choi *et al*^[142]^, found no significant difference in mortality between the use of carvedilol and bisoprolol. These two β-blockers were the most commonly described in the literature, with carvedilol prescribed in 60.5% of cases and bisoprolol in 32.7%. Nebivolol was used in 3.0% of cases, while other β-blockers, including metoprolol, betaxolol, celiprolol, atenolol, propranolol, amosulalol, bevantolol, and sotalol, accounted for 3.8%.

New emerging studies suggest the use of new combinations of drugs in patients with ADHF, such as landiolol combined with the inotrope levosimendan, which was suggested in a study by Dabrowski *et al* who stated that this combination improved cardiac function, normalized stroke volume, and allowed for a reduction in norepinephrine dosing^[136]^. Moreover, a study conducted by Ditali *et al* demonstrated that landiolol, when used with inotropes, optimizes hemodynamics and controls tachyarrhythmias without worsening hypotension in ADHF patients with hemodynamic instability^[143]^. Additionally, Chalkias *et al* and Iwahashi *et al* mentioned that landiolol effectively mediates the inotropic effect and improves hemodynamics in critically ill ADHF patients and didn’t worsen HF nor increase remodeling, with Iwahashi *et al* also reporting fewer major adverse events in patients with smaller left ventricular volumes and higher blood pressure^[144,145]^. Furthermore, Shiga *et al* assessed the role of intravenous landiolol in controlling heart rate in patients with atrial tachyarrhythmias and ADHF. His results emphasize quick action and the reversibility of potential adverse effects^[146]^. These results present a possible role for landiolol in future use in ADHF. Making it a promising therapy for stabilizing patients before initiating long-term HF management (Fig. 5).

However, the use of beta-blockers in ADHF is still in question. Further studies and randomized controlled trials are needed to form a clearer idea about how safe it is to use these drugs in different settings and how to adjust the dose in each clinical scenario.

## Non-pharmacologic approaches and emerging therapies

Despite advances in pharmacological management, a subset of patients with AHF remains refractory to conventional therapies, such as diuretics and vasodilators. These patients, particularly those who are diuretic-resistant or experience persistent congestion, face limited therapeutic options. In response, device-based interventions have emerged as a promising frontier, targeting specific physiological mechanisms to alleviate congestion, improve cardiac function, and enhance patient outcomes.

This section explores investigational devices categorized by the DRI2P2S framework. These devices leverage innovative strategies, from modulating venous capacitance and promoting fluid removal to enhancing renal perfusion and addressing interstitial congestion. By examining their mechanisms of action, clinical evidence, and potential roles in AHF management, this review aims to highlight their therapeutic promise while acknowledging the challenges and gaps that remain^[147]^.

### Dilators: expanding venous and arterial capacitance in AHF

Venous capacitance is decreased as a result of increased sympathetic tone in AHF^[148]^. Notably, splanchnic vein vasoconstriction in AHF leads to a shift of blood from unstressed to stressed vessels, thereby increasing systemic and pulmonary venous pressures^[149]^. This pathophysiology occurs in clinically symptomatic patients before any weight gain is noticed^[148]^.

Similar to vasodilators such as nitroprusside and nitroglycerin, which can redirect blood volume into the splanchnic vein, a catheter-based splanchnic vein modulation is under investigation that works by increasing both venous and arterial capacitance^[150]^.

In a pilot study of 11 patients with New York Heart Association class III/IV symptoms, reduced ejection fraction, and PCWP >15 mmHg, temporarily blocking the splanchnic nerves bilaterally resulted in symptomatic improvement without causing hypotension, as depicted by a reduction in mean arterial pressure and mean pulmonary arterial pressure^[151]^.

**Figure 6.**
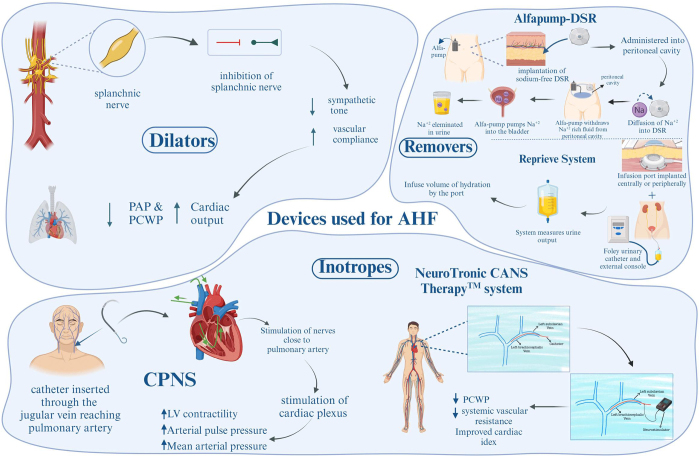
Devices utilized in AHF management: focus on dilators, inotropes, and removers. This figure showcases key devices employed in the treatment of AHF, emphasizing their mechanisms of action. Dilators, by inhibiting the splanchnic nerve, enhance vascular compliance and improve cardiac output. Inotropes, such as CPNS and NeuroTronic CANS Therapy™ systems, stimulate cardiac function and reduce pulmonary pressures. Removers, including the Alfapump-DSR and reprieve system, effectively manage fluid and sodium levels to alleviate congestion and enhance patient outcomes. Each device addresses distinct pathophysiological aspects of AHF to optimize clinical intervention.

The Splanchnic-HF-2 study tested the role of splanchnic nerve blockade in attenuating increased filling pressures after exercise in patients with chronic HF (Table 4). This clinical trial on 15 patients showed a decrease in exercise-induced pulmonary arterial pressure and PCWP and improvement in cardiac output after splanchnic nerve modulation (Fig. 6)^[152]^.

### Removers: addressing fluid and sodium overload

As patients with HF have water and sodium overload, the role of diuretics is essential in their management. However, some patients don’t experience clinical improvement after diuretic use; these diuretic-resistant cases need alternatives for diuresis, such as direct sodium removal (DSR), which has been tested by some novel devices. One of the devices in this field, the alfapump-DSR (Sequana Medical NV, Belgium), is inserted subcutaneously in the abdomen. A subcutaneously implanted port delivers sodium-free DSR infusate to the peritoneal cavity. After sodium diffuses into the DSR infusate over a predetermined time, the alfapump pumps the extracted sodium into the bladder, where it is eliminated in the urine^[153]^. This mechanism has been tested in two clinical trials: RED DESERT in eight euvolemic patients (clinical trial registration: NCT04116034) and SAHARA in 12 hypervolemic patients (clinical trial registration: NCT04882358) (Table 4). Serial DSR with diuretic removal was feasible and safe, with persistent and significant improvement along with improvement in prognostic cardiorenal parameters such as NT-proBNP, IL-6, CA-125, and GDF-15^[154]^.

Another device, the Reprieve System (Reprieve Cardiovascular, Milford, MA, USA), works by decongesting patients with ADHF. The device’s goal is to achieve a fluid balance target. Using an infusion port that can be accessed centrally or peripherally and a Foley urinary catheter connected to an external console, the system measures urine output and adjusts the infusion volume of hydration accordingly to reach a fluid balance rate^[147]^. The Reprieve system was used in 19 patients with congestive HF in the first trial and preexisting poor renal function in the second, according to two non-randomized clinical trials called TARGET-1 and TARGET-2 (NCT03897842). Patients showed a decrease in central venous pressure (CVP), improvement in renal function, and weight loss during hospitalization^[155]^. All patients found the Reprieve system to be safe and comfortable to use (Fig. 6).

### Inotropes: enhancing cardiac contractility

The sympathetic and para SNS both contribute to the cardiac plexus of nerves. Without altering heart rate, autonomic nerve stimulation raises cardiac contractility, mean arterial pressure, and myocardial relaxation during diastole^[156]^. This effect could be achieved by inserting an electrode in the pulmonary arteries that will stimulate surrounding autonomic nerves to achieve the desired effect^[156]^. Using this concept, some devices have been tested. A first-in-human, proof-of-concept study was conducted in seven patients (NCT04814134) using a catheter-based cardiopulmonary nerve stimulation (CPNS, Cardionomic Inc., New Brighton, MN). For up to 5 days, individuals with ADHF received endovascular stimulation via the CPNS by inserting a 16-French catheter percutaneously through the right jugular vein, positioning it in the right pulmonary artery. Results showed increased LV contractility by 58%, arterial pulse pressure by 20%, and mean arterial pressure by 7% without affecting heart rate^[156]^.

Another system, the NeuroTronik CANS Therapy™ System, uses an electrical stimulation catheter placed via left subclavian vein access percutaneously in the left brachiocephalic vein. Then, a neurostimulator is connected to the catheter to deliver autonomic nerve stimulation for up to 96 hours. This concept was tested in studies (NCT03169803, NCT02880683, and NCT03542123). At the transcatheter cardiovascular therapeutics symposium in 2019^[157]^, results from 12 patients with congestive HF with EF <40% showed improved CI by 22% and a decrease in PCWP by 28% and systemic vascular resistance by 22% (Fig. 6).

### Interstitial modulators: facilitating lymphatic drainage

One of the consequences of HF is reduced lymphatic drainage of edema in the interstitial space from the periphery, lungs, and abdominal organs. This underestimated pathophysiology appears to play a role in congestion since lymphatic drainage is essential for interstitial fluid removal^[158]^.

The WhiteSwell™ therapy system (WhiteSwell, Ireland) is a new device used to achieve complete decongestion without affecting renal function. The device is composed of two compliant balloons positioned at the bifurcation of the jugular and innominate veins. The system facilitates the drainage of the thoracic duct by reducing venous pressures in the thoracic duct outflow area through inflation of the balloons^[147]^. The device has been studied in a sheep model^[159]^. Results showed decreased extravascular lung water volume. Another case study on an 82-year-old woman (NCT02863796) reported positive early signals, with improvements in CVP, serum creatinine, and NT-proBNP.

**Figure 7.**
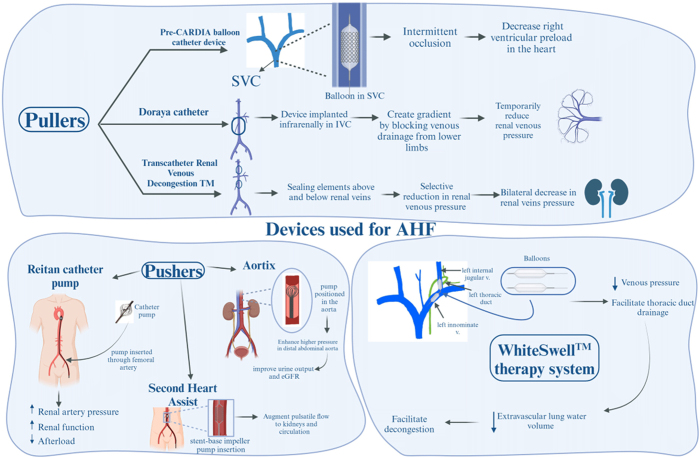
Devices utilized in AHF management: focus on pushers, pullers, and selective therapies. This figure presents various advanced devices designed for AHF management. Pushers, including the Reitan catheter pump, Aortix device, and Second Heart Assist, enhance renal perfusion, cardiac output, and afterload reduction. Pullers, such as the pre-CARDIA balloon catheter and Doraya catheter, improve venous decompression and optimize hemodynamics. Selective therapies like Fenoldopam target renal perfusion to reduce vascular resistance and prevent nephropathy. The WhiteSell™ therapy system facilitates thoracic duct drainage to manage extravascular lung water volume effectively. These devices demonstrate innovative approaches to mitigating AHF-related challenges and improving patient outcomes.

Another proposed approach for interstitial fluid and sodium removal is the AquaPass system. By increasing the skin temperature of the lower body, the AquaPass system enhances water and fluid removal without affecting core temperature. A study (NCT04578353) of the AquaPass system included 10 HF patients and 6 healthy subjects who took 3 treatment sessions for up to 4 hours. The study showed increased skin temperature without raising core body temperature, with a median weight loss of 219 ± 67 g/h and no noticeable changes in heart rate, systolic, or diastolic pressure. However, further studies are needed to evaluate the impact on outcomes (Fig. 7)^[160]^.

### Pushers: improving renal perfusion through augmented pressure

Pushers are devices used to increase renal arterial pressure to enhance renal output in ADHF patients, as low cardiac output leads to decreased renal perfusion, depicted by cardiorenal syndrome pathophysiology^[161]^.

The Reitan catheter pump (Cardiobridge, Germany) is inserted through the femoral artery percutaneously and positioned in the descending aorta to create a pressure gradient of around 10 mmHg by rotating the propeller in the pump. By creating this gradient, the device increases renal artery pressure, improves renal function, and reduces afterload, as shown by a prospective observational study on 18 patients^[162]^.

The Aortix (Procyrion, Inc., Houston, TX, USA) is an axial flow pump positioned in the aorta above the renal artery to enhance higher pressure in the distal abdominal aorta, thus augmenting renal function. This device was studied in 6 patients with renal dysfunction, showing a 10-fold improvement in urine output and enhanced eGFR^[163]^.

The Second Heart Assist (Second Heart Assist, Salt Lake City, UT, USA) provides augmented pulsatile flow to the kidneys and circulation by inserting the stent-based impeller pump in the distal aorta via femoral artery access percutaneously^[147]^. No clinical data are available yet, but the device appears promising (Fig. 7).

### Pullers: reducing preload and congestion

These devices reduce filling pressures and volume overload at the SVC, IVC, or abdominal cavity. The preCARDIA balloon catheter device (Abiomed, Danvers, MA, USA), controlled by a pump, is located in the SVC. It functions by intermittent occlusion to decrease RV preload, aiding in heart decongestion^[147]^. The VENUS-HF early feasibility, prospective study^[164]^ (NCT03836079). Patients treated for 12–24 hours demonstrated a 34% reduction in right atrial pressure, a 27% decrease in PCWP, a 130% increase in urine output over 24 hours, and a 156% rise in net fluid output. Various devices have been investigated to reduce kidney congestion. The Doraya catheter (Revamp Medical Ltd., 5 Mefi, Netania, Israel) is implanted infrarenally in the IVC, creating a controllable gradient below the renal veins, temporarily reducing renal venous pressure while briefly obstructing venous outflow from the lower limbs. The Doraya device was shown to improve clinical signs of congestion, like dyspnea, and diuretic resistance in two cases^[165,166]^.

The Transcatheter Renal Venous Decongestion™ (TRVD) System (Magenta Medical, Kadima, Israel) bilaterally decreases pressure in the renal veins by using two sealing elements in the IVC above and below the renal veins, selectively reducing renal venous pressure. No clinical trial data on drug effectiveness are available (Fig. 7)^[147]^.

### Selective infusion devices: targeting renal vascular resistance

Tubular hypoxia is one of the kidney function impairments in HF patients, caused by imposed vasoconstriction and decreased renal perfusion^[167]^. Selective infusion of a D1 receptor agonist (fenoldopam) into the kidneys to decrease renal vascular resistance has been tested using the Benephit catheter (Angiodynamics, Latham, NY), a bifurcated catheter inserted through the femoral artery percutaneously in both renal arteries^[168]^. In a post-market registry, the Benephit catheter administered fenoldopam in 501 high-risk patients for contrast-induced nephropathy, showing a lower incidence of contrast-induced nephropathy^[169]^; however, it has not been specifically tested for HF (Fig. 7).

### Temporary mechanical circulatory support: bridging the gap in cardiogenic shock management

Special devices for temporary mechanical circulatory support (TMCS) have been used and studied for cardiogenic shock management. The selection of these devices depends on many factors, including the severity of the condition, the patient’s specific shock phenotype (left, right, or biventricular failure), and available resources. These devices aim to improve tissue perfusion, support organ recovery, and achieve hemodynamic stabilization^[170]^.

One of the widely used devices for its safety and simplicity is the intra-aortic balloon pump (IABP). The IABP enhances coronary perfusion and reduces afterload through counterpulsation by inflating the balloon during early diastole and deflating just before systole^[171]^. However, the device provides only a modest cardiac output increase up to 1 L/min, making it unsuitable for severe cardiogenic shock or significant aortic regurgitation^[171]^. Furthermore, the IABP does not lower mortality in acute myocardial infarction-induced cardiogenic shock (AMI-CS), according to the IABP-SHOCK II trial^[172]^. For advanced left ventricular support, Impella devices (CP and 5.5) have emerged as preferred options. These devices improve cardiac output (up to 5.5 L/min) by directly unloading the left ventricle through an axial flow pump^[173]^. While the Impella 5.5 provides longer support and more rehabilitative possibilities^[174]^, the Impella CP is appropriate for short-term stabilization. Although these devices are successful, their use is more specialized due to the risks of hemolysis, vascular problems, and the need for fluoroscopic insertion, especially with the Impella CP^[173]^.

In cases of severe cardiogenic shock requiring both cardiac and oxygenation support, venovenous extracorporeal membrane oxygenation is a crucial option. The device can be rapidly employed and provides up to 7 L/min of flow to ensure systemic perfusion, bypassing the heart and lungs and thus providing full cardiopulmonary support^[175]^. Limitations of this device include increased left ventricular afterload, which can lead to complications like pulmonary edema^[176]^. Adjunctive venting strategies, such as atrial septostomy or Impella use, are required^[177]^.

The TandemHeart device offers both circulatory support and effective left ventricular unloading. Its use is limited to centers with specialized expertise because it requires transseptal puncture. By using a centrifugal pump, the device provides up to 6 L/min of left atrium-to-aorta flow^[178]^. It can also be used in right-sided layouts when RV assistance is required, according to recent reports^[170]^. For RV support, Impella RP and Protek Duo devices can be used. The Impella RP provides right atrium-to-pulmonary artery flow via single venous access but is contraindicated in patients with high pulmonary vascular resistance^[179]^. The Protek Duo device can be used for both oxygenation and RV support by a dual-lumen cannula, making it ideal for more complex cases^[180]^.

## Controversies and challenges in acute HF management

AHF represents a complex syndrome needing urgent and highly individualized intervention. However, management is further encumbered by several controversies surrounding the available therapies and the coming modes of treatment. The most prominent ones are the use of high-dose diuretics, early introduction of inotropes, fluid management strategies, biomarker-guided therapies, and the incorporation of novel treatments. Each of the techniques or modalities has its indications and risks, therefore underlining the need for an individualistic approach and further studies.

### High-dose diuretics: balancing benefits and risks

High-dose diuretics remain the cornerstone in the management of AHF, providing symptomatic relief for congestion, dyspnea, and pulmonary edema^[181]^. The rationale behind using high doses is the expedient removal of fluid to stabilize patients in acute decompensated states of the disease. Regrettably, this approach is also not without considerable downsides^[182]^.

Excessive dosing with diuretics is associated with a high propensity for electrolyte disturbances, including hypokalemia, hypomagnesemia, and hyponatremia, which may further contribute to arrhythmias and adversely affect overall outcomes^[183]^. These risks are compounded in patients with comorbid renal dysfunction, who may be more vulnerable to acute kidney injury. Most of them have a problem with resistance to therapy, whereby an inability to increase doses is incapable of yielding an appropriate response; this usually requires additional treatments such as the use of thiazide diuretics or the use of vasopressin antagonists^[184]^.

The chronic risks include activation of the RAAS and SNS, both of which are known to contribute to disease progression in HF ^[185]^. The available observational studies and trials, including the DOSE-AHF study, have provided data supporting the fact that higher-dose therapy has no significant mortality benefit compared to standard doses (Table 4)^[186]^.

Other strategies that have more recently come into play for reducing these risks include continuous infusion of diuretics to maintain steady plasma concentrations and hence minimize toxicity. Biomarker-guided diuretic titration is gaining attention for helping clinicians balance efficacy with safety^[187]^.

### Early inotrope initiation: weighing short-term gains against long-term risks

Inotropes represent a critical therapeutic modality in the management of patients with AHF who present with low-output syndromes, ranging from mild systolic dysfunction to cardiogenic shock^[188]^. Inotropic agents increase myocardial contractility and consequent improvement in cardiac output, symptoms of hypoperfusion, and hemodynamic stability in critically ill patients. However, the routine early initiation of inotropes in AHF management remains controversial^[189]^.

Inotropes like dobutamine and milrinone show nearly immediate symptomatic improvement; however, administration is associated with great risk^[190]^. These include arrhythmias, myocardial ischemia, and hypotension, which could worsen the disease further. Chronic inotropic therapy also increases mortality rates, especially among patients who have stable hemodynamics and do not require such aggressive levels of treatment^[191]^.

The current guidelines suggest the use of inotropes primarily in patients with severe systolic dysfunction and end-organ hypoperfusion that is unresponsive to other therapies, such as diuretics and vasodilators. However, optimal timing and duration of inotrope therapy remain active areas of investigation^[192]^.

These include, for example, emerging agents like omecamtiv mecarbil, a selective cardiac myosin activator, and levosimendan, which acts by calcium sensitization with additional vasodilating properties, offering better safety than their classical counterparts. Clinical trials are studying these agents as new options for utilizing inotropic therapy in AHF^[193]^.

### Fluid management: diuretics, ultrafiltration, and beyond

Among the cardinal features of pathophysiology, congestion is one aspect, and hence fluid management is effective and important in AHF. Though diuretics remain the cornerstone, limitations to diuretic therapy do exist- factors such as diuretic resistance, hence alternative modalities such as ultrafiltration and novel pharmacological interventions must be used^[194]^.

Ultrafiltration is a mechanical modality of fluid removal, permitting an accurate control of fluid balance that may be particularly useful in patients with severe congestion or not responsive to diuretics^[195]^. However, practical problems, such as cost, specialized equipment, and possible complications related to vascular access, thrombosis, and infection, prevent its routine application. Results of some trials, like CARRESS-HF, have shown mixed results and support further evidence toward its use in the management of AHF^[196]^.

Pharmacological alternatives represented by vasopressin antagonists and SGLT2 inhibitors are promising in improving diuresis with a relative reduction in the adverse effects of traditional diuretics (Table 4)^[197]^. Research into the lymphatic modulation therapies that aim at an improvement in lymphatic drainage and a reduction in systemic congestion represents a novel frontier in fluid management.

### Biomarker-guided therapies: a double-edged sword

Biomarkers have revolutionized the diagnosis and prognosis of AHF, providing insights not only on disease severity but also on treatment response and long-term outcomes. It is for this reason that the natriuretic peptides, especially BNP and NT-proBNP, are particularly useful biomarkers regarding volume status and the guidance of therapy. Nevertheless, the application of therapeutic decision-making is challenging^[198]^.

Although biomarker-guided approaches have the potential to individualize care and optimize outcomes, their reliability is not uniform^[199]^. Biomarker levels can be affected by several variables such as renal dysfunction, obesity, and age, which can lead to misinterpretation with overtreatment or undertreatment. In addition, thresholds for intervention have not yet been standardized for clinical practice^[200]^.

These emerging biomarkers, such as galectin-3, ST2, and mid-regional pro-adrenomedullin (MR-proADM), hold promise for further refinement of risk stratification and guiding therapy^[201]^. Their combination at the bedside with clinical and imaging data may further enhance their predictive power, thus opening new avenues toward razor-sharp evidence-based management strategies.

### Sodium-glucose cotransporter-2 inhibitors

Sodium-glucose cotransporter-2 (SGLT-2) inhibitors have gained prominence as a therapeutic option in HF, demonstrating cardiovascular benefits including reduced hospitalizations and mortality. Initially developed for diabetes, their role in acute HF remains under investigation^[202]^. Trials such as SOLOIST-WHF and STRONG-HF indicate their potential benefits, though concerns about initiating these agents during acute episodes persist. These agents are being closely studied for their safety and efficacy in reducing adverse outcomes in AHF^[203,204]^.

### Adjunctive therapies: acetazolamide and iron supplementation

Adjunctive therapies like acetazolamide and iron supplementation are emerging as valuable additions to AHF management^[205]^. Acetazolamide, a carbonic anhydrase inhibitor, has been shown in the ADVOR trial to enhance diuresis and address metabolic alkalosis, though its routine use is limited by concerns about electrolyte imbalances and renal function^[206]^. Similarly, intravenous iron supplementation has demonstrated improvements in functional capacity and quality of life in chronic HF ^[207]^, as evidenced by trials like FAIR-HF and AFFIRM-AHF^[208,209]^. However, its role during acute decompensation is less clear, necessitating cautious application

### Rapid sequence initiation of medications

Early initiation of guideline-directed medical therapy (GDMT) during hospitalization is a critical strategy for improving outcomes in AHF. Trials such as STRONG-HF, TRANSITION, and PIONEER-HF underscore the benefits of rapid up-titration of GDMT, including reductions in NT-proBNP levels and hospital readmissions^[203,210]^. Despite these advantages, challenges persist, such as medication complexity and patient tolerance, necessitating careful consideration of timing and patient stability^[211]^.

### Beta-blocker continuation during acute episodes

The continuation of beta-blockers during AHF episodes is another debated area. Evidence from trials like B-CONVINCED and OPTIMIZE-HF supports their ongoing use, showing no delays in clinical improvement and reduced mortality and readmissions^[212,213]^. However, in cases of significant hemodynamic instability, temporary withholding may be necessary, emphasizing the need for clinical judgment.

### Use of invasive hemodynamic monitoring

Invasive hemodynamic monitoring, such as pulmonary artery catheterization, provides detailed data but is controversial due to unclear survival benefits and potential adverse events^[213]^. The ESCAPE trial highlights its limited utility, recommending selective use in patients with complex or refractory HF. Similarly, ultrafiltration, although effective for diuretic-resistant volume overload, faces limitations due to resource demands and complications, as noted in trials like CARRESS-HF and UNLOAD^[214]^.

### New frontiers: challenges in implementing novel therapies

The rapid landscape for AHF is changing, with novel drugs and devices offering some hope of improving outcomes in this patient group^[215]^. SGLT2 inhibitors, developed originally for the treatment of diabetes, have been found to confer significant benefits in HF, including reduced hospitalization and mortality. However, in the acute setting, their role remains under examination, and several important logistical questions remain to be settled^[216]^.

Other frontiers in the management of AHF include device-based therapies: LVADs, pulmonary artery pressure monitors, and implantable sensors. Although novel devices have shown benefit in further improving hemodynamic monitoring and symptom control, widespread adoption of such therapies is limited by high costs, technical complexities, and specialized training^[217]^.

Precision medicine, based on genetic and proteomic data, is trying to create treatment targets that are specific for the individual patient to offer personalized management of AHF^[218]^. However, broad implementation faces high hurdles: cost, infrastructure, and integration into models of existing care.

### Multidisciplinary care: bridging the gaps

Given the multidimensional nature of AHF, management involves a multidisciplinary approach that should include cardiology, nephrology, pulmonology, and even palliative care. These models can manage the highly diversified challenges with AHF, including matters such as fluid optimization and commonly encountered comorbidities like diabetes, CKD, or COPD^[219]^.

Technology, decision-support tools, and AI all make valuable contributions to the enhancement of care delivery. AI algorithms analyze large datasets to predict clinical deterioration, provide guidance on therapy, and tailor interventions. Clinician and patient education will be critical in support of better adherence to the evidence-based practice and in engaging in shared decision-making^[220]^.

### Timing of discharge and readmission rates

Lastly, the timing of discharge and strategies to reduce readmission rates remain critical considerations. Prolonged hospitalization increases healthcare costs and exposes patients to hospital-related complications, while premature discharge may heighten the risk of readmission. Individualized discharge planning based on patient condition and clinical stability is essential to striking the right balance.

In summary, AHF management demands a nuanced and dynamic approach, integrating established therapies with emerging innovations. Ongoing research, multidisciplinary collaboration, and individualized care are pivotal to advancing outcomes for this complex syndrome.

## Future directions in research and clinical practice

The future of AHF management is characterized by major advances in research and clinical practice, with new therapies and strategies opening possibilities for a response to the challenges posed. SGLT2 inhibitors, one of which is dapagliflozin, have emerged as promising agents in HF treatment^[221]^. Other recent publications, including a retrospective propensity score-matched cohort study, have also demonstrated that dapagliflozin reduces readmission rates and the administration of loop diuretics in patients with AHF^[222]^. The DICTATE-AHF trial follows in this vein by studying the safety and efficacy of this drug in hospitalized AHF patients, but the real talking point is its potential utility^[223]^. They yield variable results and, therefore, must be taken into consideration while calling for further research into the role of SGLT2 inhibitors in acute settings.

Novel diuretic formulations are being developed in parallel, along with efficacy enhancement and improved compliance. A nasal spray formulation of the powerful loop diuretic bumetanide has been shown to possess promise for providing effective congestion relief in AHF, with the additional benefits of convenience and a noninvasive means of administration compared to traditional methods of delivery^[224]^. This can make quite a difference in the outcomes and compliance of the patients. Further, the investigation of nitric oxide pathway vasodilators, vericiguat, and inhaled nitrites is a part of establishing their role in the reduction of afterload and improvement of hemodynamics, thus offering new options for AHF management^[225]^.

Omecamtiv mecarbil is a manipulator of cardiac myosin that ushers in a novel mechanism of enhancing myocardial contractility without increasing oxygen demand. Studies were presented at a late-breaking clinical trial session at ACC18^[226]^. Trials such as GALACTIC-HF have proved their effectiveness in chronic HF, yet currently ongoing studies aim to confirm their utility during the acute phase. Similarly, opening a completely new era is the field of regenerative medicine^[227]^. Gene editing using CRISPR/Cas9 or stem cell-based therapies could repair the damaged myocardium and might reverse the signs of HF. Clinical trials such as DREAM-HF have indicated that it may be possible to ameliorate cardiac function and reduce inflammation by the injection of stem cells^[228,229]^.

Biomarker-driven therapies are also very much in vogue, with newer biomarkers like ST2, galectin-3, and MR-proADM allowing better risk stratification and therapeutic decisions. Parallel developments involve AI-driven algorithms that integrate biomarker information with clinical data to predict decompensation and guide early interventions^[230,231]^.

Telemedicine and remote monitoring are revolutionizing care with the ability to transmit real-time physiological data through wearable devices and sensors, such as the CardioMEMS HF System, which demonstrated significant reductions in hospitalizations for patients suffering from HF ^[232]^. Advanced cardiac MRI with strain imaging is yielding more precise myocardial structure and function assessments^[233]^. These tools have become important contributors to significant advances in clinical practice, helping clinicians better phenotype patients with HF, whether the cause is ischemic or non-ischemic earlier signs of decompensation. In addition to these, emerging therapies targeting molecular pathways include RNA-based treatments. An example is the RNA-based treatment for HF from Novo Nordisk, which targets myocardial remodeling and hypertrophy and is in Phase 2 testing^[234]^.

Comorbidities are, therefore, becoming an integral part of AHF management. SGLT2 inhibitors showed multifaceted benefits in the reduction of hyperglycemia, fluid overload, and cardiovascular events^[235]^. Weight loss medications, like tirzepatide, showed efficiency in the improvement of symptoms of HF in obese patients, again pointing to the fact that a holistic approach toward treatment is important^[236]^.

Meanwhile, mechanical circulatory assistance devices, such as the BiVACOR total artificial heart, and pulmonary artery pressure monitoring systems are extending the armamentarium in the management of advanced HF, offering solutions where options have been limited. Several trials are currently underway that will shape the future of AHF care^[237]^. The STRONG-HF trial investigates the effects of rapid up-titration of guideline-directed therapies during hospitalization^[238]^, while the TRANSFORM-HF trial compares torsemide and furosemide for the best diuretic in HF ^[239]^. RNA-based therapies, tested in the REVERSE-HF study as a method of reversing myocardial remodeling^[240]^, represent the increasing focus on new modalities of treatment. In summary, the future management of AHF will epitomize the integration of pharmacological innovation, regenerative medicine, precision tools, and digital health technologies with the care gaps in the current environment to realize value for the patients. This translation into practice cannot be done without continuous collaboration by researchers, clinicians, and industry stakeholders to realize a better future for those affected by AHF.

## Conclusion

AHF is a complex syndrome, and management presents significant challenges due to the complex pathophysiology and unacceptably high morbidity and mortality. In this review, mechanisms related to AHF exacerbation, limitations of current therapies, and controversies in approaches such as the use of high-dose diuretics and the use of inotropes have been touched upon. Biomarker-guided therapies, novel fluid management techniques, and new pharmacologic agents, including the use of SGLT2 inhibitors, are important, emerging strategies, but additional research is needed to optimize their appropriate utilization. Future directions highlight that regenerative medicine, RNA-based therapies, and advanced remote monitoring technologies may lead to game-changing innovation for AHF care. Personalised medicine, when combined with multidisciplinary approaches, offers the prospect of improving outcomes by addressing unique patient needs. With the increasingly heavy burden of AHF, innovation and collaboration among clinicians, researchers, and policy makers are needed. Only by further developing the treatment strategies and closing gaps in care will more effective and compassionate management for this challenging condition be accomplished.

## Data Availability

Not applicable. No original datasets were generated or analyzed for this review.

## Research registration unique identifying number (UIN)

Not applicable (narrative review; no prospectively enrolled human subjects).
